# Metabolic Alteration and Amyotrophic Lateral Sclerosis Outcome: A Systematic Review

**DOI:** 10.3389/fneur.2019.01205

**Published:** 2019-11-20

**Authors:** Mariana Dutra Brito, Gustavo Ferro Gomes da Silva, Erick Mutti Tilieri, Beatriz Grisolia Araujo, Michele Longoni Calió, Tatiana Rosado Rosenstock

**Affiliations:** ^1^Department of Physiological Science, Santa Casa de São Paulo School of Medical Science, São Paulo, Brazil; ^2^Department of Physiology, Federal University of São Paulo, São Paulo, Brazil

**Keywords:** amyotrophic lateral sclerosis, patients, metabolism, prognostic factor, systematic review

## Abstract

**Background:** The development of strategies that could not only efficiently detect the onset of Amyotrophic Lateral Sclerosis (ALS), a fatal neurodegenerative disorder with no cure but also predict its development and evaluate therapeutic intervention would be of great value. In this respect, the metabolic status of ALS patients has called attention. Hence, this study aimed to investigate the potential correlation between changes in ALS's metabolic parameters with the disease outcome in a systematic review.

**Methods:** The manuscripts were manually searched within different databases (PubMed, Web of Science and Cochrane). The inclusion criteria were original articles and reviews about individuals with ALS and its survival, disease prognosis and metabolism (weight, cholesterol, hypertension, BMI, and glycaemia). The authors also established three different exclusion criteria: studies including ALS and other degenerative disorders, works including animal models and published before the year 2000.

**Results:** In total, 29 papers were selected. From all manuscripts, only 82.8% ensured the participation of sALS patients. Also, 27.6% of selected studies described the presence of a genetic mutation. Regarding ALS prognosis, patient's age, the age of ALS onset, ALS duration and survival, <50% of the papers addressed these issues. Specifically, regarding metabolism, 65.5% of articles mentioned BMI, 20.7% mentioned any data concerning hypertension, 6.89% cardiovascular risk, 10.3% obesity, 13.78% diabetes and 10.3% glycaemia. Concerning lipid metabolism, more results were gathered, but still, they did not suffice to establish a correlation with ALS development.

**Conclusions:** Altogether, the authors concluded that available information is not enough to establish a link between ALS and metabolism. In reality, less than half of the manuscripts evaluated show an association between both factors. Nonetheless, it is worth mentioning that metabolism does influence ALS, but not in a unique manner. There is a debate about patients' hypo- and hypermetabolism. Thus, to provide a reliable record, a public policy in which all research and clinical centers might assess the parameters discussed herein is suggested. Accordingly, this systematic review attempts to provide a comprehensible database to facilitate multicentered collaboration, validation, and clinical translation.

## Introduction

The Amyotrophic Lateral Sclerosis (ALS) is a progressive and fatal neurodegenerative disorder (1–3). ALS, also known as Lou Gehrig's disease, has an incidence of 2.5 cases per 100,000 people/year and a prevalence of 4-6:100,000; definitely, it is the most common motor neuron disorder (4). Therefore, ALS has not being considered a rare disease once its developing risk is 1/400-1/700 (5). Currently, it is well-known for the involvement of more than 24 genes in ALS. However, these mutations account for 68% of familial cases (fALS) and just for 11% of sporadic ALS (sALS) (6). Oddly, 90% of all ALS cases are sporadic. ALS onset, regardless of its form, occurs in the fifth decade of life. The survival rate is 3–5 years after diagnosis, being men more affected than women (1.5:1) (7).

Along ALS occurs a specific and progressive degeneration of upper motor neurons of the corticospinal tract, motor cortex, and motor neurons from the lower brainstem and spinal cord (8–12). However, ALS is a multifactorial disorder and, consequently, not only neurons are afflicted, but also it presents reactive astrocytes, dysfunctional oligodendrocytes and activated microglia committed (13–20). Moreover, several mechanisms seem to be related to the onset and the evolution of ALS, including excitotoxicity, oxidative stress, mitochondrial dysfunction, protein aggregation, genetic mutations, diminishment in the axonal transport, modifications in RNA metabolism, and neuroinflammation (21–32).

Notably, there is neither a cure for ALS, nor even an effective therapy, although there are drugs used to attenuate ALS' symptoms and bring significant benefits to patients, such as antioxidants, anti-inflammatory, antiapoptotic, and anticytotoxic agents (4, 25, 33). To date, one of the most used medications in the USA is Rilutek™ (riluzole), a glutamate release inhibitor (34). Several clinical trials have demonstrated an increase in the survival rate of ALS patients after Rilutek™ (35–37). More recently, the U.S. Food and Drug Administration (FDA) approved Radicava™ (edaravone), a powerful antioxidant that is not restricted to eliminate hydroxyl radicals and reactive oxygen but counteracts the increase in prostacyclin production (38). Because these new findings are promising, attention has been focused on prognostic factors and biomarkers for ALS. Indeed, biomarkers discovery may enable a reliable diagnosis and a predictable follow-up; nowadays, ALS's diagnosis and prognosis are mainly based on physical exams (39). Furthermore, the discrepancies of signs among patients interpret clinical trials somewhat dubious (40).

A variety of clinical prognostic factors and several ALS-related biological biomarkers have been listed (41–45), including some associated with metabolism (42–58). With interest, metabolic changes in ALS animal models were also observed (46, 59–61). However, there is no consensus on whether these metabolic parameters are indeed related to the disorder prognosis itself or just represent a specific dataset from a single-center and population. In this context, lipid content, cholesterol, and BMI are often contradicting. Additionally, genetic mutation, glycaemia, hypertension, TGL, LDL, HDL, and even medication are seldom mentioned in ALS manuscripts, which make it more difficult to collect clinical investigation outcomes.

Thus, to investigate the potential correlation of metabolic status with ALS's outcome and survival, authors have decided to put together recent data regarding BMI, hypertension, cardiovascular risk, obesity, diabetic, glycaemia, hyperlipidaemia, triglycerides (TGL), LDL, HDL, and cholesterol. In addition, further information directly and indirectly related to metabolism were included, such as the age of onset, smoking habits, disease duration (years), survival (years), population/ethnic group, mutation, family members with ALS (%), family members with motor disorders (n), first motor signs (%), ALSFRS and ALS medication (including medicine intake period). Therefore, this study presents a systematic review regarding the possible relationship between ALS subjects' survival and the course of the disease, with metabolic-related factors.

## Methods

### Eligibility Criteria

**Figure 1:**
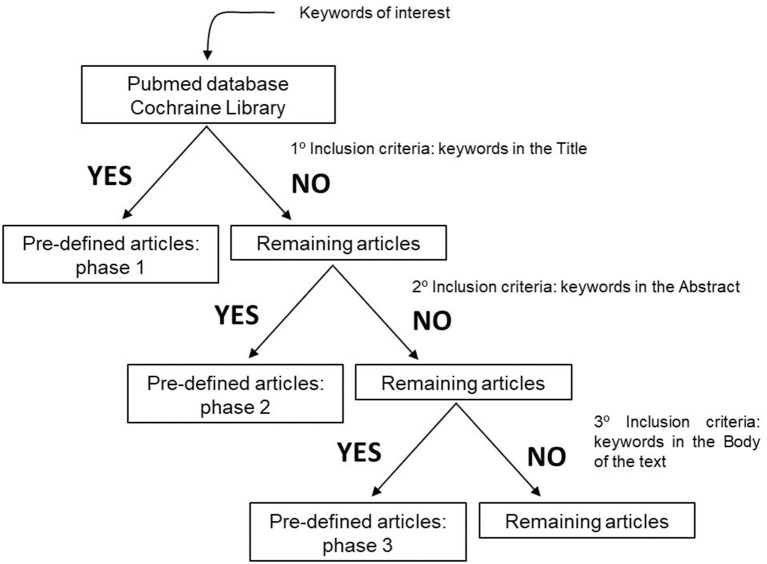
Inclusion criteria used to define the articles that would be evaluated and submitted to this systematic review.

**Figure 2:**
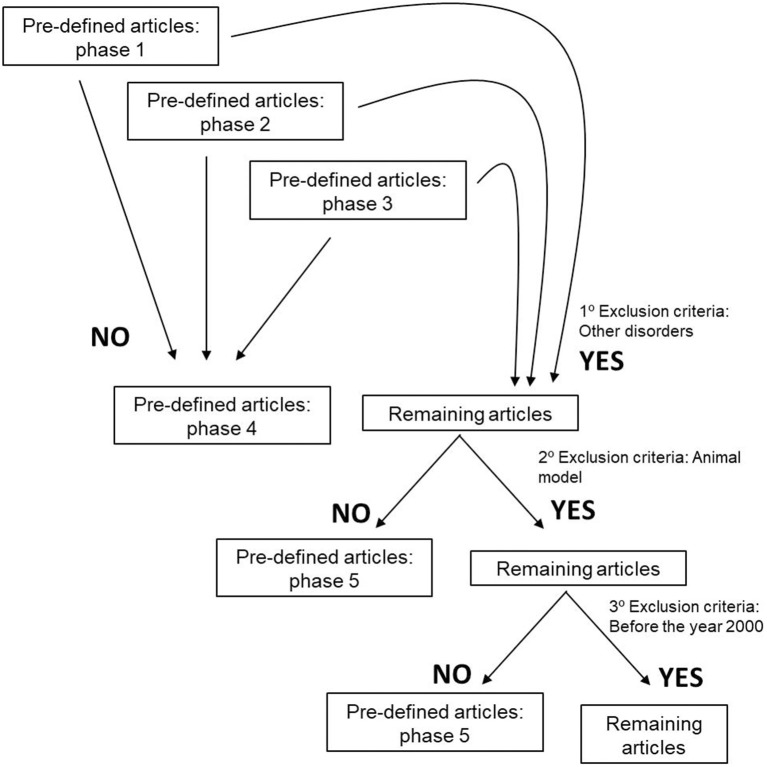
Exclusion criteria used to define the articles that would be evaluated and submitted to this systematic review.

To perform our systematic review, we established inclusions and exclusion criteria. Clear inclusion criteria were original articles and reviews concerning ALS patients and their survival, disease prognosis and metabolism (Figure 1). Five different exclusion criteria were endorsed: (i) studies including ALS and other degenerative disorders, such as Alzheimer disease, Frontotemporal dementia, Myotonic Dystrophy, and Muscular Atrophy, (ii) articles including animal models, (iii) manuscripts published before the year 2000, due to the fact that several papers are issued every year, and epidemiology approaches and biochemical methodologies are constantly changing. Importantly, authors also excluded, (iv) studies in which there is a mix of ALS-FDT patients and ALS-AD subjects without any kind of discrimination, and (v) articles that despite having the keyword of our research is about guidance on the management and care of ALS patients (Figure 2).

### Search Strategy

The keywords and expression of interest were manually searched in various databases, i.e., PubMed, Web of Science and Cochrane. The databanks were selected for topics assurance and for including journals of authors' interest. Specifically, the following strategy was used to establish the inclusion/exclusion criteria: (i) “Amyotrophic Lateral Sclerosis” (not the term ALS) AND “patient” AND “cohort” AND “mutation” AND “mitochondria” OR “metabolism”; (ii) Amyotrophic Lateral Sclerosis” AND “patient” AND “cohort” AND “aging” AND “mitochondria” OR “metabolism”; (iii) “Amyotrophic Lateral Sclerosis” AND “patient” AND “cohort” AND “mutation” AND “glycaemia” OR “hypertension” OR “weight” OR “cholesterol”; (iv) “Amyotrophic Lateral Sclerosis” AND “patient” AND “cohort” AND “aging” AND “glycaemia” OR “hypertension” OR “weight” OR “cholesterol”; (v) “Amyotrophic Lateral Sclerosis” AND “patient” AND “cohort” AND “mutation” AND “aging” AND “metabolism” OR “mitochondria”; (vi) “Amyotrophic Lateral Sclerosis” AND “patient” AND “cohort” AND “mutation” AND “aging” AND “glycaemia” OR “hypertension” OR “weight” OR “cholesterol”; (vii) “Amyotrophic Lateral Sclerosis” AND “patient” AND “glycaemia” AND “weight” OR “hypertension” OR “cholesterol”; (viii) “Amyotrophic Lateral Sclerosis” AND “patient” AND “weight” AND “hypertension” OR “cholesterol”; (ix) “Amyotrophic Lateral Sclerosis” AND “patient” AND “hypertension” AND “cholesterol”; (x) “Amyotrophic Lateral Sclerosis” AND “patient” AND “glycaemia” AND “weight” AND “hypertension” OR “cholesterol”; (xi) “Amyotrophic Lateral Sclerosis” AND “patient” AND “glycaemia” AND “hypertension” AND “cholesterol”; (xii) “Amyotrophic Lateral Sclerosis” AND “patient” AND “weight” AND “hypertension” AND “cholesterol”; (xiii) “Amyotrophic Lateral Sclerosis” AND “patient” AND “glycaemia” AND “weight” AND “hypertension” AND “cholesterol”; (xiv) “Amyotrophic Lateral Sclerosis” AND “patient” AND “cohort” AND “glycaemia” OR “weight” OR “hypertension” OR “cholesterol”; (xv) “Amyotrophic Lateral Sclerosis” AND “patient” AND “cohort” AND “glycaemia” OR “weight” OR “hypertension” OR “cholesterol” AND “mitochondria” OR “metabolism.”

### Outcome

Data outcome was categorized as follows: year of publication, total number of ALS patients (n), familial ALS (fALS) patients (n), sporadic ALS (sALS) patients (n), male (M)/female (F) (%) (ratio), El Escorial, age of subject (years), age of onset (symptoms), disease duration (years), survival (years), population/ethnic group, mutation, relatives with ALS (%), relatives with motor disorders (n), first motor signs (%), ALSFRS, BMI, smoking, hypertension, cardiovascular risk, obesity, diabetic, glycaemia, hyperlipidaemia, triglycerides (TGL), LDL, HDL, cholesterol, ALS medication, and treatment extent.

### Risk of Bias

The authors did not perform any assessment for the risk of bias. Such tools are used mainly for randomized controlled trials (RCTs), and consequently, this tool would not be appropriate. As an alternative, data was collected and the limitation of each study was recorded. The results are presented herein.

### Statistical Analysis

Results for descriptive analyses were expressed in absolute numbers (*n*) and percentages (%), and the data acquired were demonstrated in different tables. No sufficient data were gathered to perform a meaningful meta-analysis.

## Results

The search resulted in the collection of 924 manuscripts from PubMed, 54 from Web of Science and 52 from Cochrane (including original articles and reviews). After applying inclusion and exclusion criteria (Figures 1, 2), followed by a more in-depth and comprehensive evaluation of the pre-selected articles, 29 articles were considered for the study. Tables 1–**8** summarize the collected data. As we can observe, from all manuscripts, 17.2% excluded fALS patients, which means that 82.8% of all articles ensured the participation of sALS subjects only. Besides, 93.1% of the studies reported M/F ratio. Despite the significant percentage of studies mentioning this parameter, discrepancies among reported M/F were noticed (Table 1).

**Table 1:** Number of ALS patients per study, the percentage of SALs and FALs patients, and the distribution of ALS subjects between sex in general population [male (M)/female (F) ratio].

**References**	**Total of ALS patients (*n*)**	**ALSf patients (*n*)**	**ALSs patients (*n*)**	**Male (M)/Female (F) (%) (ratio)**
Chaussenot et al. (62)	106	26	80	N/A
Chiò et al. (48, 49)	658	N/A	N/A	50.7 (M)/49.3 (F) (1:1)
Chiò et al. (41)	638	N/A	N/A	55.2 (M)/44.8 (F) (1.2:1)
Dedic et al. (63)	82	all excluded	82	46.2 (M)/53.8 (F) (0.85:1)
Delaye et al. (64)	30	N/A	N/A	50 (M)/50 (F) (1:1)
Dorst et al. (43)	486	16	470	80.3 (M)/19.7 (F) (4:1)
Dupuis et al. (42)	369	N/A	N/A	52 (M)/40 (F) (1.3:1)
Golomb et al. (65)	10	N/A	N/A	60 (M)/40 (F) (1.5:1)
Hollinger et al. (66)	1,439	N/A	N/A	60 (M)/40 (F) (1.5:1)
Huang et al. (67)	413	All excluded	413	58.3 (M)/41.7 (F) (1.4:1)
Huisman et al. (68)	674	All excluded	674	62 (M)/38 (F) (1.6:1)
Kasarskis et al. (69)	80	N/A	N/A	65 (M)/35 (F) (1.8:1)
Korner et al. (70)	514	N/A	N/A	56 (M)/44 (F) (1.3:1)
Li et al. (71)	294	23	271	71 (M)/29 (F) (2.4:1)
Mandrioli et al. (72)	2,354	138	2216	55.1 (M)/44.9 (F) 1.2:1)
Mandrioli et al. (73)	275	N/A	N/A	55.6 (M)/44.4 (F) (1.2:1)
Mariosa et al. (74)	636,132	N/A	N/A	51.2 (M)/48.8 (F) (1:1)
Millecamps et al. (75)	162	162	All excluded	61.7 (M)/38.3 (F) 1.6:1)
Miller et al. (76)	21	21	All excluded	N/A
Moglia et al. (77)	650	39	617	55.4 (M)/44.6 (F) (1.2:1)
Moreau et al. (78)	120	N/A	N/A	56 (M)/46 (F) (1.2:1)
Mouzat et al. (79)	438	N/A	N/A	56.1 (M)/43.9 (F) (1.3:1)
Nieves et al. (80)	302	N/A	N/A	58.9 (M)/41.1 (F) (1.4:1)
Nunes et al. (81)	37	N/A	N/A	48.6 (M)/51.4 (F) 0.9:1)
Rafiq et al. (82)	512	17	495	64.6 (M)/35.4 (F) (1.8:1)
Shefner et al. (83)	13 (placebo)	N/A	N/A	54 (M)/46 (F) (1.2:1)
Sutedja et al. (51)	334–303	All excluded	334–303	57 (M)/43 (F) (1.3:1)
Zinman et al. (84)	164	N/A	N/A	60 (M)/40 (F) (1.5:1)
Wei et al. (85)	450	All excluded	450	57.9 (M)/42.1 (F) (1.4:1)

To better understand ALS prognosis, information regarding fALS and mutations were assembled; amongst which, the most cited were SOD1, FUS, TARDBP, VAPB, ANG, C9orf72, and CHCHD10 (Table 2). However, only 62.5% of the articles certified the inclusion of fALS patients only (27.6% of all selected studies described the genetic mutation).

**Table 2:** Identification of ALS mutations and the percentage of ALS patients' relatives with ALS and motor disorders.

**References**	**Mutation**	**Relatives with ALS (%)**	**Relatives with motor disorders (*n*)**
Chaussenot et al. (62)	CHCHD10	N/A	N/A
Chiò et al. (48, 49)	N/A	N/A	N/A
Chiò et al. (41)	N/A	N/A	N/A
Dedic et al. (63)	N/A	N/A	0
Delaye et al. (64)	N/A	N/A	N/A
Dorst et al. (43)	N/A	N/A	N/A
Dupuis et al. (42)	N/A	N/A	N/A
Golomb et al. (65)	N/A	N/A	1 (PD)
Hollinger et al. (66)	N/A	55	N/A
Huang et al. (67)	N/A	N/A	N/A
Huisman et al. (68)	N/A	0	N/A
Kasarskis et al. (69)	N/A	N/A	N/A
Korner et al. (70)	N/A	N/A	N/A
Li et al. (71)	CHCHD10	N/A	N/A
Mandrioli et al. (72)	N/A	N/A	N/A
Mandrioli et al. (73)	N/A	N/A	N/A
Mariosa et al. (74)	N/A	N/A	N/A
Millecamps et al. (75)	SOD1, FUS, TARDBP, VAPB, ANG	N/A	N/A
Miller et al. (76)	SOD1	4.70	N/A
Moglia et al. (77)	C9orf72	N/A	N/A
Moreau et al. (78)	N/A	N/A	N/A
Mouzat et al. (79)	N/A	N/A	N/A
Nieves et al. (80)	N/A	N/A	N/A
Nunes et al. (81)	N/A	N/A	N/A
Rafiq et al. (82)	N/A	N/A	N/A
Shefner et al. (83)	N/A	N/A	N/A
Sutedja et al. (51)	N/A	N/A	N/A
Zinman et al. (84)	N/A	8	N/A
Wei et al. (85)	N/A	N/A	N/A

To further evaluate ALS symptoms, as well as ALS prognosis, patient's age, the age of ALS onset, ALS duration, and survival, data were tabulated in Table 3. The authors could observe that 58.6% of the manuscripts specified the age of ALS onset (mean 59.8 years). For ALS duration and survival, 31 and 44.8% of studies, respectively, addressed this issue. Precisely, the mean of ALS duration is about 2.15 years, while the mean of ALS survival is 2.88 years. Moreover, to investigate the influence of ethnicity on ALS prognosis, few data were acquired; only two studies mentioned the word “caucasian” and only one mentioned the word “white.” No information is known regarding the socio-economic status of ALS individuals.

**Table 3:** El Escorial and general data of ALS patients: age, age of onset (symptoms), disease duration (years), survival (years), population/ethnic group, and socio-economic status.

**References**	**El Escorial**	**Age (years)**	**Age of onset**	**Disease duration (years)**	**Survival (years)**	**Ethnic group**	**Socio-economic status**
Chaussenot et al. (62)	Yes	65.6	62.4	N/A	N/A	French	N/A
Chiò et al. (48, 49)	Yes	N/A	61.8	N/A	N/A	Italian	N/A
Chiò et al. (41)	Yes	N/A	66.3	N/A	1.7	Italian	N/A
Dedic et al. (63)	Yes	N/A	53.78	N/A	4.19	Serbians	N/A
Delaye et al. (64)	Yes	N/A	66.5	0.42	N/A	French	N/A
Dorst et al. (43)	Yes	N/A	57.6	N/A	4.25	German	N/A
Dupuis et al. (42)	Yes	57.5	N/A	N/A	1	French	N/A
Golomb et al. (65)	N/A	N/A	61.7	N/A	N/A	American	N/A
Hollinger et al. (66)	N/A	N/A	60.1	2	2.1	Caucasian (57.4%)	N/A
Huang et al. (67)	Yes	51.8	50.3	1.8	3.1	Chinese	N/A
Huisman et al. (68)	Yes	N/A	62.4	N/A	N/A	Dutch	N/A
Kasarskis et al. (69)	Yes	58.7	N/A	N/A	N/A	American	N/A
Korner et al. (70)	Yes	58.8	N/A	N/A	3.5	German	N/A
Li et al. (71)	Yes	N/A	49	N/A	N/A	Chinese	N/A
Mandrioli et al. (72)	Yes	N/A	64.21	N/A	3.6	Italian	N/A
Mandrioli et al. (73)	Yes	N/A	65.2	N/A	N/A	Italian	N/A
Mariosa et al. (74)	N/A	53	N/A	N/A	1	Swedish (85%)	N/A
Millecamps et al. (75)	Yes	53	N/A	4.4	4.6	Caucasian	N/A
Miller et al. (76)	N/A	48.8	N/A	N/A	N/A	American (white 87.5%)	N/A
Moglia et al. (77)	Yes	N/A	66.4	N/A	3.6	Italian	N/A
Moreau et al. (78)	N/A	N/A	62.7	N/A	2.5	French	N/A
Mouzat et al. (79)	Yes	N/A	61.9	2.9	N/A	Caucasian	N/A
Nieves et al. (80)	Yes	63.2	N/A	N/A	N/A	American	N/A
Nunes et al. (81)	Yes	69	N/A	N/A	N/A	French	N/A
Rafiq et al. (82)	Yes	55	N/A	2.3	N/A	European	N/A
Shefner et al. (83)	Yes	53	N/A	1.08	N/A	American	N/A
Sutedja et al. (51)	Yes	60–64	N/A	3.5–2.6	3.5–2.6	Dutch	N/A
Zinman et al. (84)	Yes	63.7	N/A	N/A	N/A	Canadian	N/A
Wei et al. (85)	Yes	55.4	54.5	1.48	N/A	Chinese	N/A
Mean		57.84	59.82	2.15	2.88		

*The table also shows the mean of ALS age, age of onset, duration of ALS and survival*.

Considering that the motor symptoms' onset, as well as ALS's phenotypic features, can be related to the disease development and brain pathology, first motor signs and ALSFRS-R were also evaluated (Table 4). The most prevalent motor alteration among ALS patients was related to the upper or lower limbs (71.66%), followed by symptoms occurring at the bulbar level (28%). Specifically, about monitoring ALS progression, 48.3% of the studies used ALSFRS-R; the mean ratio of it was 36.4.

**Table 4:** First motor signs of ALS patients and ALSFRS scale.

**References**	**First motor signs (%)**	**ALSFRS**
Chaussenot et al. (62)	N/A	N/A
Chiò et al. (48, 49)	69.4 (S)/30.6 (B)	30.2
Chiò et al. (41)	N/A	37.4
Dedic et al. (63)	63.4 (S)/36.6 (B)	40.6
Delaye et al. (64)	47 (S)/53 (B)	30.1
Dorst et al. (43)	81.7 (L)/18.3 (B)	36.2
Dupuis et al. (42)	75 (L)/25 (B)	N/A
Golomb et al. (65)	N/A	N/A
Hollinger et al. (66)	N/A	N/A
Huang et al. (67)	77.7 (S)/22.3 (B)	31.2
Huisman et al. (68)	N/A	N/A
Kasarskis et al. (69)	72.5 (L)/26.3 (B)/1.2 (general)	36.1
Korner et al. (70)	72 (S)/28 (B)	N/A
Li et al. (71)	N/A	N/A
Mandrioli et al. (72)	66 (S)/24 (B)	N/A
Mandrioli et al. (73)	69.8 (S)/30.2 (B)	N/A
Mariosa et al. (74)	N/A	N/A
Millecamps et al. (75)	93 (S)/7 (B)	N/A
Miller et al. (76)	95.3 (L)/4.7 (B)	N/A
Moglia et al. (77)	68.8 (L)/31.2 (B)	40.5
Moreau et al. (78)	73 (S)/29 (B)	37
Mouzat et al. (79)	69 (L)/31 (B)	N/A
Nieves et al. (80)	71.5 (S)/27.7 (B)	37
Nunes et al. (81)	40.5 (S)/59.5 (B)	N/A
Rafiq et al. (82)	79.3 (L)/19.7 (B)	38.6
Shefner et al. (83)	N/A	38.4
Sutedja et al. (51)	73–70 (S)/27–30 (B)	N/A
Zinman et al. (84)	70 (S)/30 (B)	37.7
Wei et al. (85)	79 (L)/21 (B)	39.1
Mean	71.66 (S-L)/28 (B)	36.4

*The table also shows the mean of the most predominant motor symptoms and the mean of ALSFRS*.

Concerning smoking habits (Table 5), only 17.2% of the evaluated manuscripts pointed out this factor among individuals with ALS. Explicitly, just one study excluded all smokers from statistics. About metabolism (Tables 5, 6), 65.5% of articles mentioned BMI (mean, 24.4). Moreover, only 20.7% declared any data in the matter of hypertension, 6.89% of cardiovascular risk, 10.3% of obesity, 13.78% of diabetes and 10.3% of glycaemia (Table 5). Regarding lipid metabolism (Table 6), more results were collected. Specifically, 31, 34.48, 34.48, and 41.4%, of the manuscripts show, respectively, TGL (mean, 4.13 mmol/L), LDL (mean, 5.40 mmol/L), HDL (mean, 2.21 mmol/L), and cholesterol (mean, 23.25 mmol/L). But, this information did not suffice to establish a correlation between ALS development and/or progression and metabolism alterations. In fact, 48.28% of the manuscripts evaluated show an association between factors (Table 7).

**Table 5:** Smoking habits, BMI and metabolic parameters of ALS patients.

**References**	**Smoking**	**BMI**	**Hypertension**	**Cardio vascular risk**	**Obesity**	**Diabetic**	**Glycaemia**
Chaussenot et al. (62)	N/A	N/A	N/A	N/A	N/A	N/A	N/A
Chiò et al. (48, 49)	N/A	25.1	N/A	N/A	N/A	N/A	N/A
Chiò et al. (41)	N/A	24.5	N/A	N/A	N/A	N/A	N/A
Dedic et al. (63)	N/A	26.74	N/A	N/A	N/A	N/A	N/A
Delaye et al. (64)	0	23.7	N/A	N/A	N/A	N/A	N/A
Dorst et al. (43)	N/A	25.4	N/A	N/A	N/A	9.70%	6.23 mmol/L
Dupuis et al. (42)	N/A	24.6	N/A	N/A	N/A	N/A	N/A
Golomb et al. (65)	N/A	N/A	N/A	N/A	N/A	N/A	N/A
Hollinger et al. (66)	N/A	N/A	36.90%	N/A	9.10%	9%	N/A
Huang et al. (67)	N/A	21	N/A	N/A	N/A	N/A	N/A
Huisman et al. (68)	19.70%	25.7	N/A	N/A	N/A	N/A	N/A
Kasarskis et al. (69)	All excluded	27.1	All excluded	N/A	All excluded	All excluded	N/A
Korner et al. (70)	N/A	N/A	31.50%	N/A	N/A	N/A	N/A
Li et al. (71)	N/A	N/A	N/A	N/A	N/A	N/A	N/A
Mandrioli et al. (72)	N/A	24	N/A	N/A	N/A	N/A	N/A
Mandrioli et al. (73)	N/A	24.5 #	N/A	N/A	N/A	N/A	5.05 mmol/L
Mariosa et al. (74)	N/A	N/A	N/A	N/A	N/A	N/A	4.98 mmol/L
Millecamps et al. (75)	N/A	N/A	N/A	N/A	N/A	N/A	N/A
Miller et al. (76)	N/A	N/A	N/A	N/A	N/A	N/A	N/A
Moglia et al. (77)	N/A	24.3	45.50%	71.10%	N/A	9.10%	N/A
Moreau et al. (78)	26%	N/A	57%	N/A	20%	9%	N/A
Mouzat et al. (79)	N/A	N/A	N/A	N/A	N/A	N/A	N/A
Nieves et al. (80)	N/A	26	N/A	N/A	N/A	N/A	N/A
Nunes et al. (81)	N/A	21.6	N/A	N/A	N/A	N/A	N/A
Rafiq et al. (82)	N/A	24.7	N/A	N/A	N/A	N/A	N/A
Shefner et al. (83)	N/A	25.1	N/A	N/A	N/A	N/A	N/A
Sutedja et al. (51)	17%c−43%f	25	26%	24% £	46%	5%	N/A
Zinman et al. (84)	N/A	25.4	N/A	N/A	N/A	N/A	N/A
Wei et al. (85)	31%	22	16.20%	N/A	N/A	N/A	N/A
Mean		24.4					

*The table also shows the mean of BMI. Some parameters found within the evaluated population were cardiovascular risk (£, events related), smoking habits (c, current or f, former), and BMI (#, baseline)*.

**Table 6:** Blood tests outcome of ALS patients.

**References**	**TGL**	**LDL**	**HDL**	**Cholesterol**	**Hyperlipidemia**
Chaussenot et al. (62)	N/A	N/A	N/A	N/A	N/A
Chiò et al. (48, 49)	6.38 mmol/L	7.14 mmol/L	3.29 mmol/L	11.71 mmol/L	N/A
Chiò et al. (41)	N/A	N/A	N/A	N/A	N/A
Dedic et al. (63)	1.87 mmol/L	2.95 mmol/L	1.37 mmol/L	5.8 mmol/L	N/A
Delaye et al. (64)	N/A	3.64 mmol/L	1.56 mmol/L	6.51 mmol/L	N/A
Dorst et al. (43)	1.77 mmol/L	3.87 mmol/L	1.29 mmol/L	6 mmol/L	N/A
Dupuis et al. (42)	10.06 mmol/L	12.39 mmol/L	4.64 mmol/L	193.62 mmol/L	N/A
Golomb et al. (65)	N/A	N/A	N/A	N/A	N/A
Hollinger et al. (66)	N/A	N/A	N/A	N/A	26.30%
Huang et al. (67)	7.07 mmol/L	5.99 mmol/L	2.56 mmol/L	11.23 mmol/L	N/A
Huisman et al. (68)	N/A	N/A	N/A	N/A	N/A
Kasarskis et al. (69)	N/A	N/A	N/A	N/A	All excluded
Korner et al. (70)	N/A	N/A	N/A	N/A	N/A
Li et al. (71)	N/A	N/A	N/A	N/A	N/A
Mandrioli et al. (72)	N/A	N/A	N/A	N/A	N/A
Mandrioli et al. (73)	5.5 mmol/L	7.21 mmol/L	2.7 mmol/L	10.98 mmol/L	N/A
Mariosa et al. (74)	1.33 mmol/L	3.69 mmol/L	1.52 mmol/L	5.58 mmol/L	N/A
Millecamps et al. (75)	N/A	N/A	N/A	N/A	N/A
Miller et al. (76)	N/A	N/A	N/A	N/A	N/A
Moglia et al. (77)	N/A	N/A	N/A	N/A	N/A
Moreau et al. (78)	N/A	N/A	N/A	(32%)	N/A
Mouzat et al. (79)	N/A	N/A	N/A	N/A	N/A
Nieves et al. (80)	N/A	N/A	N/A	N/A	N/A
Nunes et al. (81)	N/A	N/A	N/A	11.32 mmol/L	N/A
Rafiq et al. (82)	1.6 mmol/L	3.8 mmol/L	1.5 mmol/L	5.9 mmol/L	N/A
Shefner et al. (83)	N/A	N/A	N/A	N/A	N/A
Sutedja et al. (51)	N/A	3.3 mmol/L	1.7 mmol/L	5.7 mmol/L	N/A
Zinman et al. (84)	N/A	N/A	N/A	N/A	N/A
Wei et al. (85)	1.6 mmol/L	N/A	N/A	4.7 mmol/L	N/A
MEAN	4.13 mmol/L	5.40 mmol/L	2.21 mmol/L	23.25 mmol/L	

**Table 7:** Summary of the correlation between metabolic parameters and ALS course and prognosis.

**References**	**Presence of association**	**No association**	**N/A**	**Metabolic parameter**
Chaussenot et al. (62)			✓	
Chiò et al. (48, 49)		✓		Hyperlipidemia
Chiò et al. (41)	✓			Creatinine levels
Dedic et al. (63)		✓		Hyperlipidemia
Delaye et al. (64)	✓			Dyslipidemia
Dorst et al. (43)	✓			Elevated triglyceride and cholesterol
Dupuis et al. (42)	✓			Hyperlipidemia
Golomb et al. (65)			✓	
Hollinger et al. (66)	✓			Antecedent hypertension and hyperlipidemia
Huang et al. (67)		✓		Total cholesterol, TG, LDL or the LDL/HDL
Huisman et al. (68)	✓			Low premorbid BMI and a high fat intake
Kasarskis et al. (69)	✓			Body composition
Korner et al. (70)		✓		Cardiovascular diseases or risk factors
Li et al. (71)			✓	
Mandrioli et al. (72)	✓			Increase of triglycerides
Mandrioli et al. (73)	✓			Hypertension and heart diseases
Mariosa et al. (74)	✓			Imbalance between apoB and apoA-I, and LDL-C and HDL-C
Millecamps et al. (75)			✓	
Miller et al. (76)			✓	
Moglia et al. (77)		✓		Hypertension, type 2 diabetes and cardiovascular risk factors
Moreau et al. (78)	✓			Chronic hypertension
Mouzat et al. (79)	✓			LXRgenes
Nieves et al. (80)			✓	
Nunes et al. (81)	✓			Higher BMI
Rafiq et al. (82)		✓		Lipid profile
Shefner et al. (83)			✓	
Sutedja et al. (51)		✓		Vascular risk factors
Zinman et al. (84)			✓	
Wei et al. (85)	✓			Higher levels of HbA1c, but not fasting blood glucose concentrations

Additionally, to understand if the data outcome could be influenced by medication intake, prescriptions to ALS subjects were also investigated (Table 8). Around 20.7% of the studies mentioned that patients were under Riluzole™ treatment. Only one study stated that patients had been taking the medicine since diagnosis. About other prescriptions, 13.8% of the articles declared that ALS individuals were under cholesterol and hyperlipidaemia lowering agent therapy, or they were taking antioxidants taking antioxidants, antihypertensive, and anti-diabetic agents.

**Table 8:** Medication taken by ALS patients.

**References**	**ALS medication**	**For how long**	**Medication (other)**
Chaussenot et al. (62)	N/A	N/A	N/A
Chiò et al. (48, 49)	Riluzole (9%)	N/A	N/A
Chiò et al. (41)	N/A	N/A	N/A
Dedic et al. (63)	N/A	N/A	N/A
Delaye et al. (64)	Riluzole (all)	From diagnosis	Tocopherol and cholesterol lowering agents
Dorst et al. (43)	N/A	N/A	Simvastatin
Dupuis et al. (42)	N/A	N/A	N/A
Golomb et al. (65)	N/A	N/A	Hyperlipidaemia lowering agent
Hollinger et al. (66)	N/A	N/A	N/A
Huang et al. (67)	Riluzol (25.1%)	N/A	N/A
Huisman et al. (68)	N/A	N/A	N/A
Kasarskis et al. (69)	N/A	N/A	N/A
Korner et al. (70)	N/A	N/A	N/A
Li et al. (71)	N/A	N/A	N/A
Mandrioli et al. (72)	Riluzole (82.41%)	N/A	N/A
Mandrioli et al. (73)	Riluzole (94.2%)	N/A	N/A
Mariosa et al. (74)	N/A	N/A	N/A
Millecamps et al. (75)	N/A	N/A	N/A
Miller et al. (76)	N/A	N/A	N/A
Moglia et al. (77)	N/A	N/A	N/A
Moreau et al. (78)	N/A	N/A	N/A
Mouzat et al. (79)	N/A	N/A	N/A
Nieves et al. (80)	N/A	N/A	N/A
Nunes et al. (81)	N/A	N/A	N/A
Rafiq et al. (82)	N/A	N/A	N/A
Shefner et al. (83)	N/A	N/A	N/A
Sutedja et al. (51)	N/A	N/A	Antihypertensive (26%), Antidiabetic (5%)
Zinman et al. (84)	Riluzole (82%)	N/A	N/A
Wei et al. (85)	N/A		N/A

## Discussion

Although several mechanisms are being related to ALS, its diagnosis occurs relatively late and, by then, 50% of neurons degenerated, and its prognosis is hard to predict (86). The lack of knowledge regarding secondary mechanisms related to the disease progression along with the limitation concerning studies enclosing individuals presenting ALS represents a struggle for the academic community. The development of strategies that could not only efficiently detect ALS onset but also predict its development and evaluate the therapeutic intervention, would be of great value. In this respect, metabolic status has called attention. Hence, this study aims to put together, in a systematic review, data from ALS patients, which is directly related to their metabolic status i.e., hypertension, cardiovascular risk, obesity, diabetic, glycaemia, hyperlipidaemia, triglycerides (TGL), LDL, HDL, and cholesterol. Further, authors recorded information related to the age of onset, disease duration (years), survival (years), population/ethnic group, mutation, relatives with ALS (%), relatives with motor disorders (*n*), first motor signs (%), ALSFRS, smoking habits, and ALS medication. Altogether, we indicated that while there is not an assertive data to establish any direct link between changes in metabolic parameters and the progression and survival of ALS, metabolism does impact on ALS. This means that some pathways can be related to it and can modulate ALS outcome, but the complete via and its regulation are not well-defined yet. Accordingly, several studies assume that it is very difficult to establish a correlation between those factors. Indeed, only 48.28% of the articles used to perform this systematic review show an association, meaning that this topic is still a matter of debate.

One of the first data that called the authors' attention due to their strangeness was the presence (or not) of genetic mutation in ALS subjects; only 6.8% of the studies excluded fALS (Table 1). This means that 93.2% of articles put together all types of ALS patients in the same group. Such data indicate that clinical studies did not take into consideration the genetic background of investigated individuals. Hence, considering all ALS individuals as equals (fALS and sALS) could generate a considerable bias.

It is known that ALS patients have a distinct outcome and different age of onset and survival rate (48, 49). For that reason, it was suggested that such differences could be associated to environmental factors that, in turn, might influence ALS genesis and duration (prognosis) (8, 87–95). Such aspects seem to include extreme physical activities, herbicide/pesticide exposure, neurotoxins, viral infection, prion disease, immune response, vascular risk factors and type 1 diabetes (8, 94, 95). On the other hand, diabetes type 2 in elderly, moderate physical activity, fat accumulation, and variations in glucose metabolism, high BMI and hypertension are described to be protective (94). Nevertheless, studies about the relationship between diabetes and ALS have produced conflicting results. In young patients, diabetes is a consistent risk factor for ALS; in older patients, diabetes protects against ALS in Europe but increases the risk of ALS in Asia. Intriguingly, both environment and genotype might subsidize this discrepancy (94, 95).

Other parameters that are often mentioned in ALS patients' studies, in an attempt to correlate ALS prognosis with survival, are patients' age, age of onset and survival rate (that is calculated considering both previous factors) (Table 3). As previously shown, half of the manuscripts depicted patients' age and the age of onset, making it hard to come to any conclusion concerning these factors. Besides, the exact meaning of “age of onset” contrast among works; in this review, two evaluated manuscripts declared its definition. Thus, future studies would be undoubtedly favored if the designation of onset was clear. Likewise, data interpretation would become easier, since results from different study centers could be comparable. Importantly, other groups also suggested that not only the onset but also sex ratio, age at diagnosis, comorbidities, and survival could be due to genetic background, predisposition, socio-economic status and patient's ethnicity (89, 94, 96–100). Actually, we consider that ethnic group is essential for the interpretation of prognostic factors, as it relies (at least partially) on genetics and because it is known that several polymorphisms are population/ethnic-dependent and can modulate metabolism (96, 101–103). Oddly, only two of the evaluated manuscripts mentioned the words “Caucasian” and “white,” indicating that the ethnicity of ALS patients may not have been evaluated. Furthermore, in most reports, the study group is from the same country as the authors. Thus, some favoritism in the interpretation of clinical trials could be assumed.

It is well-known that ALS symptoms appear according to the group of affected neurons. For this reason, one can show changes in the upper or lower limbs (known as the spinal form of ALS) and/or can present dysphagia, dysphonia, or dysarthria (symptoms correlated to the bulbar form). We demonstrated that 71.66% of ALS subjects presented the spinal form and 28% bulbar (Table 4). Although almost 30% of the manuscripts did not mention this factor, the results seem rather consistent among all research centers. Surprisingly, 48.3% of the studies monitored ALSFRS-R; such scale infers about the deterioration caused by the disorder and estimates disease progression per month (104). Considering that ALSFRS-R is world-wide accepted and that more than 50% of clinical evaluations did not cogitate them, the real estimation of ALS patients' evolution is jeopardized, indicating that conclusions regarding any parameter based on clinical studies should be reviewed.

Despite the lack of homogeneity among the examined articles, and consequently, the absence of data that could support an accurate ALS evaluation, the authors further considered the smoking habits (one of the factors that often influence metabolism and patient survival) (Table 5). As already mentioned, 17.2% of all articles included smoking habits in their analysis. Considering that ALS subjects suffer from shortness of breath and that they must be under respiratory therapy throughout their lives, smoking should be determinant to patient's quality of life, response to treatment, and survival.

Because metabolism is the central target of our review, specific parameters are shown in Tables 5–7. For BMI, 65.5% of all articles reported their values. On the other hand, hypertension, cardiovascular risk, obesity, diabetes, and glycaemia were less mentioned (<20%) (Table 5). About lipid metabolism, more results were gathered; around 35% of manuscripts described TGL, LDL, HDL and cholesterol levels (Table 6). However, available information is not enough to establish a direct link between ALS and metabolism alterations. In reality, less than half of the manuscripts evaluated show an association between both factors (Table 7). Nonetheless, it is worth mentioning that metabolism does influence ALS, but not in a unique manner. In fact, in literature, there is a debate about patients' hypo- and hypermetabolism. The so-called hypermetabolic condition is correlated with an augmentation in the energy expenditure to maintain mobility and ventilation avoiding, as a consequence, weakness and exhaustion of the remaining innervated muscles (105–108). Moreover, in an attempt to avoid an increase in oxidative stress, it is suggested that mitochondria become uncoupled ensuing in hypermetabolism (109, 110). Thus, the high resting metabolism could be activated to maintain energetic status. Because of that, hyperlipidaemia and increments in gluconeogenesis, lipolysis and ketogenesis pathways are sighted as neuroprotective (106–108, 111, 112). Interestingly, it was observed a hypolipidemia state in the pre-symptomatic ALS mouse model, in addition to changes in complex lipids during the first phase of motor symptoms (113, 114). Nonetheless, it was also reported that alterations in metabolism, exactly in the lumbar spinal cord in the SOD1G93A mice model before motor symptoms, are primarily caused by the mutation itself than a function of ALS's course (115).

However, it is important to stress that this is not a consensus in the literature and it is defiance for the knowledge of ALS neuropathology (116). Indeed, Chiò et al. described that there was not any effect of metabolism on survival (48, 49), although they suggested that dietetic habits could account for differences in phenotypes and disease progression (48, 49). This hypothesis, by the way, is in accordance with several reports that suggest that hypermetabolism seems to be interconnected with a predisposition of ALS subjects to malnutrition as a result of fear of choking and aspiration, dysphagia and inability to feed themselves (58, 117, 118). It was also shown *in vivo* FDG-PET study performed with C9orf72 mutation's patients that brain hypometabolism was consistent with ALS clinical phenotypes (119). Moreover, Cedarbaum et al. indicated that such inconsistencies may also be related to the insufficiency relevant information of ALSFRS-R subscale to estimate physical activity (120). Hence, no conclusion involving these parameters should be regarded either.

Because it is known that some medication also modifies cellular metabolism, we also considered ALS subjects' prescriptions in our analysis (Table 8). We observed that (i) 13.8% of articles declared that patients were taking antioxidants, antihypertensive and/or anti-diabetic drugs, in addition to cholesterol and hyperlipidaemia lowering agents, and (ii) 20.7% of them stated that patients were under Riluzole™ treatment (only one study specified that it has been taken since diagnosis). This is very intriguingly data since most of the patients are under a disease-modifying therapy with Riluzole. Riluzole (2-amino-6-trigluoromethoxy benzothiazole) is a wide-spectrum agent ranging from being an anti-glutamatergic drug to increase glial glutamate reuptake and to modulate post-synaptic receptor-mediated effects and excitotoxic pathways (121–126). Moreover, it is known that Riluzole influences, depending on the disease stage, distinct paths, making the intracellular signals of this drug hard to follow (127). Nevertheless, to the context of this review, it is important to mention that Riluzole can interfere with calcium buffering capacity, mitochondrial membrane potential (ΔΨm), sodium currents, voltage-dependent calcium channels and calcium-dependent potassium currents (128). Curiously, Riluzole can also act as a free radical scavenger, blocking reactive oxygen species production through electron transport chain, or by inhibition of calcium efflux at synapse sites (128). Thus, Riluzole, *per se* might change cellular metabolism.

### Limitations of the Study

Several factors might have contributed to the data generated and showed in this review. In fact, numerous methodological limitations of previous works must be listed, i.e., system used by primary centers to acquire evidence, population evaluated (representative vs. available), single-centered vs. multicentric study, sample size, study duration, gene mutation, familial vs. sporadic ALS cases, ethnicity, genetic background, environmental exposure to risk factors, incomplete ALSFRS-R (or the absence of it) and type of study (prospective vs. retrospective; the last can be inaccurate due to lack of data and even lack of patient, in addition to an indisposition of relatives to corroborate with some information) (73). Likewise, in the most of published studies, there were also significant discrepancies regarding age of onset, sex ratio and dose/time of pharmacological therapies. Still, authors believe that there is no sufficient data to perform a meaningful meta-analysis and, therefore, establish a correlation with ALS. Also, a prospective study with an in-depth examination of ALS patients could be supportive.

### Conclusion

Our review points to two main conclusions based on recent developments, (i) there is no assertive data available in the literature to establish a specific link between metabolic parameters and the progression and survival of ALS, and (ii) metabolism does influence ALS, but not in a unique manner. This means that some pathways can be related to it and can modulate ALS outcome, but the complete via and its regulation are not well-defined yet. Furthermore, our conclusions can be an alert about how researchers conduct their investigation and how epidemiology studies are designed, since the effort that is being made toward the establishment and validation of a clinical biomarker seems to occur in vain, given the data inconsistency, database divergences, and the absence, in most papers, of relevant information. Essentially, which are the factors, other than the ones mentioned herein, which should be revisited? We believe that investigators should consider exploring other factors, as food habits, neurodevelopment deficits, complications at birth and mother's infection and hypertension (all aspects already correlated to brain development and function). It is worth mentioning that all the topics above can induce modifications in cellular homeostasis through fluctuations in mitochondrial function, ATP synthesis, gene transcription and/or epigenetic regulation. Considering that all these features can also be modulated by genotype background (mutations, polymorphisms), ethnic group, behavioral habits, and environmental factors, ALS's etiology is far more complicated and heterogeneous than we presume (8, 92, 94, 95, 129). Moreover, we should also consider the role of microbiota. Indeed, it was already demonstrated in recent reports that gut microbiota modulates inflammation through short-chain fatty acids and endotoxin synthesis. The microbiota is correlated not only with Alzheimer's disease, neuromyelitis optica, multiple sclerosis, and Parkinson's disease but also ALS (130, 131).

Thus, to provide a reliable record, it is suggested a public policy in which all research and clinical centers might assess the parameters discussed herein. In accordance, Martin et al. in 2016 also recommended a collaborative study involving a wide international consortium to investigate, using a standard methodology, the link between ancestry, environment and ALS phenotype (129). Moreover, the authors genuinely suggest that all conclusions based on clinical trials and patients' evaluation to date should be reconsidered. Accordingly, this systematic review attempts to provide a comprehensible database to facilitate multicentered collaboration, validation, and ultimately, clinical translation.

## Data Availability Statement

All data generated or analyzed during this study are included in the article/supplementary material.

## Author Contributions

TR and MB analyzed all the articles' titles and abstracts. The defined articles were randomly divided by all authors (GS, MB, ET, BA, and TR), and TR and MC double read all of them. TR wrote the manuscript, and both TR and MC revised it. All authors approved the final version.

### Conflict of Interest

The authors declare that the research was conducted in the absence of any commercial or financial relationships that could be construed as a potential conflict of interest.
